# The containment of potential outbreaks triggered by imported Chikungunya cases in Italy: a cost utility epidemiological assessment of vector control measures

**DOI:** 10.1038/s41598-018-27443-9

**Published:** 2018-06-13

**Authors:** F. Trentini, P. Poletti, F. Baldacchino, A. Drago, F. Montarsi, G. Capelli, A. Rizzoli, R. Rosà, C. Rizzo, S. Merler, A. Melegaro

**Affiliations:** 1 DONDENA Centre for Research on Social Dynamics, Bocconi University, Milano, Italy; 2 Center for Information Technology, Bruno Kessler Foundation, Trento, Italy; 3 Department of Biodiversity and Molecular Ecology, Research and Innovation Centre, Fondazione Edmund Mach, San Michele all’Adige, Trento, Italy; 4 Entostudio, Ponte San Nicolò, Padova, Italy; 5 Istituto Zooprofilattico Sperimentale delle Venezie, Padova, Italy; 6 Department of Infectious Disease, Istituto Superiore di Sanità, Roma, Italy; 7 Department of Social and Political Sciences, Bocconi University, Milano, Italy

## Abstract

The arrival of infected travelers from endemic regions can trigger sustained autochthonous transmission of mosquito-borne pathogens in Europe. In 2007 a Chikungunya outbreak was observed in central Italy, mostly affecting two villages characterised by a high density of *Aedes albopictus*. The outbreak was mitigated through intervention strategies reducing the mosquito abundance. Ten years later, in 2017, sustained Chikungunya transmission was documented in both central and southern Italy. The proposed analysis identifies suitable reactive measures for the containment and mitigation of future epidemics by combining epidemiological modeling with a health economic approach, considering different arrival times of imported infections and possible delays in the notification of cases. Obtained estimates suggest that, if the first notification will occur in the middle of the mosquito breeding season, the combination of larvicides, adulticides and breeding sites removal represents the optimal strategy. In particular, we found that interventions implemented in 2007 were cost-effective, with about 3200 prevented cases, 1450 DALYs averted and €13.5 M saved. Moreover, larvicides are proven to be more cost beneficial in early summer and warmer seasons, while adulticides should be preferred in autumn and colder seasons. Our results provide useful indications supporting urgent decision-making of public health authorities in response to emerging mosquito-borne epidemics.

## Introduction

Globalisation processes and changes in climatic conditions are progressively increasing the suitability of temperate climate regions for the establishment and diffusion of mosquito species that are competent vectors for the transmission of tropical infectious diseases in human populations. This phenomenon combined with an increase in human mobility is raising major concerns on the potential diffusion in non-tropical countries of vector-borne diseases caused by arboviruses such as Zika, Dengue, West Nile and Chikungunya^1,2^.

In particular, Chikungunya virus (CHIKV) may represent a major threat for many European countries, as a consequence of the high transmissibility potential of this pathogen^3–5^, and the widespread abundance of *Aedes albopictus* in temperate climate regions, where this mosquito species represents the competent vector for CHIKV transmission^6,7^. CHIKV is characterised by a non-negligible burden of chronic sequelae potentially rising after human infection^6^, and significant costs caused by disease treatment^5,8–11^. In 2007, Italy was the first European country reporting sustained CHIKV transmission, with a major outbreak affecting two municipalities of Emilia Romagna and counting more than 200 confirmed cases^12^. The index case of the observed epidemic was a man travelling from Kerala, India, who later tested positive to the virus. Sporadic local transmission events have also been reported in France between 2010 and 2017^13,14^, triggered by the arrival of infected travelers from endemic areas. More recently, as of September 18^th^ 2017, two clusters of autochthonous CHIKV transmission were detected in the cities of Anzio and Rome, two areas located 60 km apart in the Lazio region in Italy^15^. Afterwards, an additional cluster of transmission was found in the city of Guardavalle Marina in the Calabria region^16^. The Italian authorities immediately launched epidemiological and entomological investigations in the areas and control strategies were implemented to reduce the mosquito density in the affected areas. The symptom onset for the first cases was retrospectively estimated between 26 and 27 June, i.e. three months before the first cluster was identified^17^.

No specific treatment or vaccine is yet available to prevent CHIKV infection and the containment of potential future outbreaks mainly relies on the interruption of the transmission chain by means of well-designed preventive and reactive strategies to reduce the mosquitoes density^18,19^. In the presence of autochthonous transmission, public health measures will be likely implemented to interrupt the spread of the infection. However, the arrival of imported infected cases from endemic areas seldom triggers large epidemics and may often results in only few sporadic cases. In addition, only a fraction of infections shows symptoms and is notified, so that unnoticed transmission may occur. It is therefore essential, when assessing which are the most suitable containment and mitigation measures, to explicitly account for the risk of observing large outbreaks as a consequence of the importation of new cases and also to consider all potential health outcomes and economic costs generated by the different intervention policies.

The effectiveness of vector control strategies adopted during the 2007 Italian outbreak^20^ and of larvicide treatments in areas characterised by low-moderate vector abundance^21^ has been recently investigated. Cost associated with preventive measures adopted in Emilia Romagna after the 2007 outbreak were also quantified^22^ and the impact of CHIKV either in terms of Disability Adjusted Life Years (DALYs) losses^10,23^ or economic costs was evaluated for tropical regions^5,10,24^. However, only few studies were conducted within a cost-utility framework to investigate the cost-effectiveness of vector control strategies. One recent study has focused on the cost-utility of routine larvicide applications aimed at preventing Dengue and Chikungunya transmission in temperate climate regions^25^. A second one has evaluated preventive measures to reduce the burden of Dengue in an urban setting where the disease is endemic, by taking into account insecticide resistance^26^. Finally, a web-based cost-effectiveness tool has been proposed to assess funding commitments required to reduce the burden of Zika in the Americas^27^. However, the assessment of integrated and optimal reactive strategies in response to the notification of imported cases of vector-borne infections in temperate climate countries still represents an open key issue.

The main goal of this work is to evaluate the cost-effectiveness of reactive vector control interventions aimed at reducing the potential burden and costs due to CHIKV disease, and to assess whether time of notification of the first index case has an impact on the identification of the optimal control strategy. Although the proposed analysis was informed with data and information derived from the CHIKV Emilia Romagna outbreak, the outcome of the current work is intended to have a broader application and to inform the design of future policy recommendations to face new potential CHIKV outbreaks in Europe.

## Results

Model estimates suggest that, during the 2007 outbreak, if no measure had been taken, the cost of Chikungunya cases and their burden in terms of associated morbidity would have been respectively €14.9 M and 1600 DALYs lost. The implemented interventions whose costs, around €50000, can be considered negligible with respect to the cost of illness, resulted in roughly 3200 (95% CI: 2580–3560) prevented cases, 1450 (95% CI:1,160–1,600) DALYs averted, and €13.5 M (95% CI:10.9M–15M) saved for treatment of symptomatic cases, either hospitalised or ambulatory patients. In line with estimates provided elsewhere^28^, the overall cost of the performed intervention was found to be, on average, €40 per hectare.

**Figure 1:**
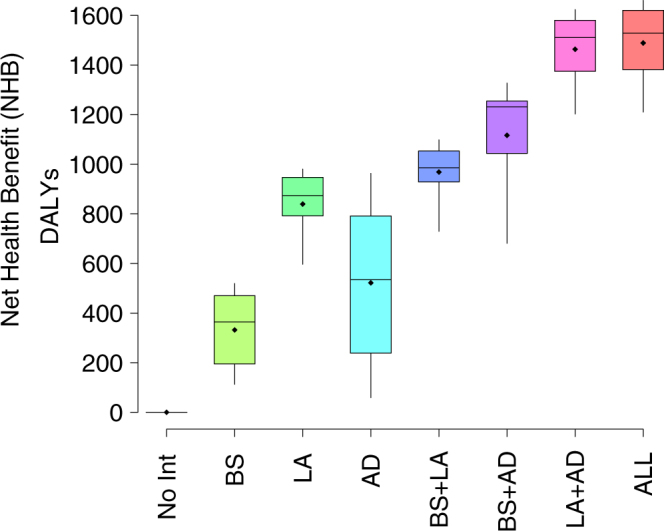
Posterior distribution of the Net Health Benefit (NHB) for each intervention strategy considered^12^ versus the hypothetical scenario where no intervention is implemented, under the assumption that the willingness to pay for the healthcare system is €30000. Intervention strategies associated with positive values of the NHB should be considered as cost-effective. BS refers to breeding sites removal, LA to larvicides and AD to adulticides, while No Int refers to the scenario without any intervention.

Combining net costs and benefits, and assuming a willingness to pay (WTP) of €30000, the Net Health Benefit (NHB) associated with the intervention performed during 2007 was found to be between 1200 and 1600 DALYs (Fig. 1). More specifically, our results suggest that whereas the use of larvicides and adulticides both contributed significantly to the cost-effectiveness of the implemented vector control program, breeding sites removal had a negligible effect on the NHB. The latter indication emerges from the small differences in NHB distribution resulting for the integrated vector control program with respect to the one obtained by considering adulticides and larvicides only.

**Figure 2:**
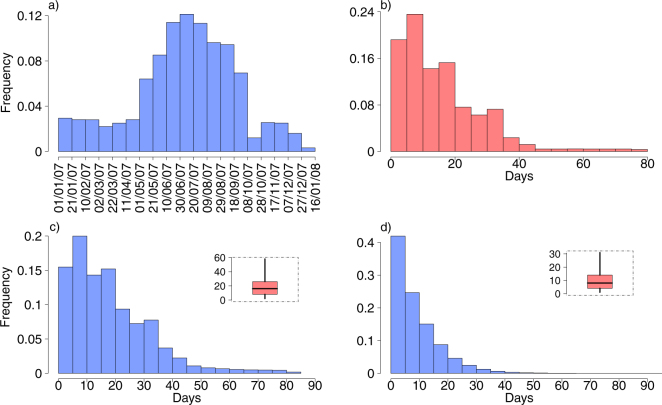
(**a**) Sampling distribution of dates of arrival of CHIKV cases from endemic area^34^. (**b**) Sampling distribution of time elapsed between the arrival and the notification of CHIKV cases to public health authorities^34^. (**c**) Posterior distribution of time elapsed between the arrival of the first case, either symptomatic or asymptomatic, and the first notification to public health authorities. (**d**) Posterior distribution of time elapsed between the observation of the last symptomatic case and the last infected vector.

The probability distributions associated with (i) different dates of arrival of CHIKV cases from endemic areas, and (ii) delays in their notification are shown in Fig. 2a and 2b. In a perfect reporting system, if the index case were symptomatic, the time at first notification would coincide with its diagnosis. However, delays in the notification of infections could arise as a consequence of both defections in the reporting system and silent transmission due to asymptomatic cases. In our model we assume that only 82% of cases are symptomatic and only 54% of symptomatic cases are notified^29^. According to our model, the delay in the notification of the first case is expected to be 18.6 days on average, ranging from 2 to 58 days (Fig. 2c). Similarly, the average number of days between the observation of the last symptomatic case and the time when the last infected vector dies is estimated to be on average 9.1 days, ranging from 0 to 31 days (Fig. 2d). These two quantities are critical for policy decision makers as the former provides important information on the onset of an epidemic, and the latter on how long should the public healthcare system wait before ceasing interventions after the observation of the last symptomatic case.

**Figure 3:**
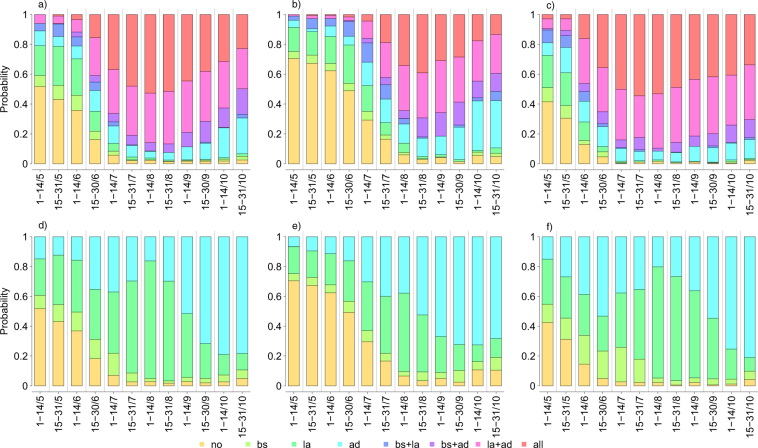
Top panels show probabilities of being the most cost effective of all possible strategies for different times at first notifications when considering temperature records observed during the Italian outbreak in 2007 (**a**), with temperatures decreased (**b**) or increased (**c**) by 1.5 °C. Bottom panels show probabilities of being the most cost effective among the considered single strategies for different times at first notifications with the observed temperature records (**d**), with temperatures decreased (**e**) or increased (**f**) by 1.5 °C

The probability of observing autochthonous transmission, as a consequence of the importation of cases, increases with mosquito density. Optimal control strategies were therefore investigated by accounting for different times at notification of the first case and the uncertainty surrounding possible chains of events affecting the CHIKV transmission dynamics and notification. These include for instance the onset of asymptomatic (unnoticed) infections and the intrinsic stochasticity characterising the transmission of the pathogen from humans to mosquitoes, and vice-versa. We found that the suitability of interventions, in terms of probability of being the most cost-effective strategy, varies broadly with time at notification of the first CHIKV case (Fig. 3a). In particular our results suggest that, in principle, if a first disease case is notified in the early spring no intervention is required. Clearly, this might not be realistically feasible in the presence of an ongoing outbreak. However, it is important to stress that, according to our model simulations, in this case the effort required to contain the spread of Chikungunya is significantly smaller than the one needed if disease cases are notified between June and October. More specifically, we found that larvicides represents the most beneficial intervention in late spring, while the combination of larvicides and adulticides alone or with breeding sites removal represents the optimal control measure during summer and in early autumn. Interestingly, the application of adulticides alone emerges among the most beneficial strategies when the first case is notified at the end of the mosquito season (which in Italy corresponds to late October) proving to be as cost-beneficial as the integrated program.

The impact of single interventions was also evaluated in order to assess which vector control activities should be prioritised if autochthonous transmission were observed along the season.

Our results suggest that the cost-effectiveness of the integrated program is mainly driven by larvicide applications when the first notification occurs between the beginning of June and the end of August (Fig. 3d). On the other hand, if a CHIKV case is reported at the beginning of September or later, adulticides prove to be the most effective intervention in terms of NHB. Breeding sites removal is instead dominated along the whole season, and should be considered only in addition to other strategies. A sensitivity analysis was carried out to investigate the robustness of obtained estimates against the uncertainty surrounding the efficacy of vector control activities in reducing the mosquito population (see Appendix SI for details). Obtained results show that a lower efficacy of single interventions decreased the expected NHB associated with the considered control programs. However, the prioritisation of different strategies at different times across the mosquito season remains substantially unchanged with respect to results of our baseline analysis. In particular, the adoption of reactive vector control measures resulted always cost effective when the notification of a first case occurs between mid June and late October.

However, when considering scenarios associated with colder temperatures (Fig. 3b) model results showed that it would be beneficial not to intervene until mid-June, while, later in the season, the use of adulticides emerge as the optimal strategy. On the other hand, when assuming higher temperatures (Fig. 3c), reactive interventions are required also for notifications occurring earlier, and the best intervention strategy will be represented either by the combination of adulticides and larvicides or by the integrated program, combining all vector control activities.

More generally, when looking at the single strategies (Fig. 3e,f), we found that adulticides should be prioritised at the very end of the season, no matter what assumption is made on the temperature levels, and in the middle or towards the end of the mosquito season in the presence of colder temperatures. On the opposite, warmer temperatures increase the benefits of larvicides, which have a more long lasting impact on the mosquito population density. However, if the first notification occurs early in the season, when autochthonous transmission is likely to die out soon, the cost effectiveness of adulticides is slightly higher due to their immediate effect on the few remaining cases and infected vectors. Further details on the sensitivity analyses are presented in Appendix S1.

## Discussion

Autochthonous CHIKV transmission represents a potential threat for temperate climate regions due to the widespread establishment of competent vectors and the constant arrival of travelers who were infected in endemic areas. The proposed analysis aims at identifying optimal and cost-effective reactive strategies to reduce the risk of large epidemics of tropical vector-borne diseases in those European regions characterised by favorable epidemiological and climatic conditions. Our model is specifically tailored to well represent the *Ae*. *albopictus* vital dynamics and CHIKV transmission in Italy, where this mosquito is the only competent vector for transmission of the infection. This means that suitable control strategies and their relative cost-effectiveness may be completely different for other vectors and diseases. In order to extend the proposed analysis to other arboviruses (such as Dengue, or Zika) or vectors (as the *Ae*. *aegypti*), assumptions made both on the vital dynamic of vectors involved, on parameters and mechanisms describing the transmission, and on the associated burden and health costs should be all carefully revised^2,4,25^.

Italy is still by far the most affected European country in terms of *Ae*. *albopictus* diffusion on the territory, with almost all provinces (97%) reporting the presence of tiger mosquitoes, mainly in the northeastern area between Alps and Appennini mountains, and on the cost of the Adriatic Sea^30^. In particular, Veneto and Emilia Romagna report the highest presence with more than 75% of municipalities affected by such vector^31^. This country has experienced two CHIKV outbreaks in the last decade and remains at high risk of future epidemics^4,17^.

Recent modeling studies, based on robust entomological surveillance data, have highlighted that the highest transmissibility potential of arboviruses in temperate climate regions is expected in small-medium size cities characterised by a high density of vectors in the human population^2,4^. This is in agreement with what has been observed during the Italian 2007 CHIKV outbreak, which has mainly affected two small municipalities in the north-eastern area (Emilia Romagna Region).

As for local transmission events recently reported in central Italy, epidemiological and entomological investigations of separate clusters of Chikungunya cases are currently ongoing. However, it has been suggested that the first date of symptoms’ onset of the reported cases was August 5, 2017 and that the first transmission event might have taken place around mid-July or before^15,17^. In agreement with these estimates, we found that the notification of cases can occur with an average delay of 19 days after their actual arrival in the area, this being due to problems in the reporting system but also the presence of asymptomatic, though infectious, cases. Our results also suggest that silent transmission may continue up to two months after the observation of the last symptomatic case and this should be taken into consideration when planning the interruption of intervention activities. These results are important for planning outbreak investigation activities and monitoring clusters of cases during the final phase of an epidemic.

Our results suggest that the combination of vector control activities carried out during the 2007 outbreak and targeting different life-stages of the mosquito were the optimal intervention strategy among all the possible combinations considered, and may have produced about 1450 DALY lost averted and €13.5 M saved. These results support the high cost-effectiveness of the performed interventions and quantify their impact both from an economic and a health perspective. Cost-effectiveness of different vector control activities, however, varies as the notification occurs in different periods of the mosquito season. Specifically, we found that, if a CHIKV case is notified during the summer, when mosquitoes density reaches its peak, the combination of larvicide and adulticide applications alone or in addition to breeding sites removal were proven to be the optimal intervention strategy. Interestingly, by disentangling the contribution of the single interventions, larvicide applications resulted more cost-effective between July and August, while our estimates suggest that adulticides should be prioritised from September till the end of the mosquito season. On the other side, the contribution of breeding sites removal to the effectiveness of a combined intervention was found negligible throughout the whole season. The prioritisation of larvicides during early phases of the mosquito season may derive from the fact that, as suggested in a previous work^18^, larvicides applications are less costly and have longer efficacy. On the opposite, adulticides applications are more expensive and, although they have an immediate impact on the density of female adults, their effects are expected to rapidly wane over time, due to the replenishment of adults from mosquito aquatic stages.

These results should be interpreted in light of the current recommendations on how mosquito control methods should be integrated into practice for improving of vector management. A recent review of the ECDC^32^ of current international, national and sub-national technical documents, guidance and recommendations on the control of invasive mosquitoes suggested that larvicide applications and the removal of *Ae*. *albopictus* breeding sites are recommended both as preventive measures and in response to an outbreak, while the use of adulticides - when permitted - is recommended only in extreme circumstances. These include the emergence of a new outbreak, but also the identification of significant risk to public health caused by a persistent high density of adult mosquitoes^32^. The importance of investing in vector control measures and their cost-effectiveness in reducing the burden of unchecked vector-borne epidemics has been already documented^27^. However, strategies based on continuous larval control have been also suggested to be counterproductive as a consequence of possible insecticide resistance^26^ and other vector control strategies, such as the use of genetically modified mosquitoes^27^, may be considered in the future as well.

Limitations of the proposed analysis mostly lie on the highly specific epidemiological and climatic conditions considered, which has been defined by mimicking temperature records and the mosquito density characterising the observed outbreak in Emilia-Romagna during 2007. In addition, our investigation is focused on the population and area mirroring the two municipalities mostly affected during the 2007 CHIKV outbreak. These conditions should be carefully considered as representative of all temperate climate regions. However, they may represent an important illustrative case of favorable climatic and epidemiological scenario for the occurrence of potential large epidemics in non-endemic areas. Furthermore, a sensitivity analysis carried out by considering alternative temperature patterns reveals that the obtained results in terms of vector control activities that should be prioritised during the season may be generalised also to areas with different climatic conditions.

The proposed analysis is based on the simplistic assumption that interventions are carried out along with the first notified infection to the public health system, which could be either an imported case or an autochthonous one, if the former was not reported. Clearly interventions at the local level may have occurred before the notification in the national register, but robust data on this are lacking. Therefore, we adopted a conservative approach relying, also, on the available sparse information which suggests this delay to be on average smaller than three days.

Strategies implemented after two or more notified infections were not considered in the carried out analysis. Nevertheless, our study is restricted to European regions, where we expect a vector control program to be adopted as soon as a CHIKV autochthonous transmission is notified to the public health authorities. Finally, the proposed analysis focuses on mitigation and containment measures in response to the notification of a CHIKV case so that our findings should not be considered to design routine prevention protocols^25^.

Nonetheless, obtained results give important insights, from a policy-making perspective, on the prioritisation of different vector control activities that should be implemented at different time points during the mosquito breeding season in response to notifications of imported infectious cases from countries where vector borne diseases are endemic.

## Methods

### Epidemiological model

A stochastic mathematical model is used to mimic the transmission mechanism characterising the CHIKV virus, taking into account the seasonal variation in *Ae*. *albopictus* abundance within an illustrative mosquito season (between April-October), and to simulate the impact of different vector control activities. The model is the one developed and calibrated in Poletti and coauthors^3^, here used to evaluate the effects of intervention strategies in response to the notification of a CHIKV case returning from an endemic region. Details on the model assumptions and on the considered vector control strategies can be found in the Appendix S1.

Briefly, the transmission of CHIKV is simulated in a human population of approximately 4,000 individuals, mimicking the epidemiological conditions observed during the 2007 Italian outbreak, assuming both air and water temperature experienced in that favorable season.

Parameters driving the efficacy of the simulated vector control activities were taken from the original analysis^3^. Breeding sites removal are assumed to reduce by 40% eggs, larvae and pupae, and decrease the carrying capacity associated with the aquatic stages by 40% for 30 days. The reduction of larvae and adults through larvicides and adulticide applications are respectively assumed to be 90% and 95%. While adulticides are effective within one day, the reduction of larvae determined by larvicides is assumed to persist for one month after the intervention is carried out. Efficacies of different interventions were assumed according to what reported in the guidelines of the European Chemicals Agency (ECHA)^33^. However we conducted a sensitivity analysis to test the robustness of model estimates with respect to possible uncertainty on the assumed efficacy of different vector control activities. In this case, we sampled the efficacy of breeding sites removal uniformly between 20–60%, and the efficacy of larvicide and adulticide applications between 60–95% (see Appendix S1 for detailed information).

The performed analyses are based on the simulation of different arrival times and notification periods as informed with data on the importation of CHIKV cases in Italy over the last 10 years^34^. These sources of uncertainty, combined with the seasonal variation in vector abundance driven by temperature patterns, result in a variety of possible outcomes triggered by the importation of an infected human case. In the absence of vector control measures, the simulated transmission dynamics can result in: (i) the recovery of the index case with no additional human infections; (ii) the occurrence of few sporadic (possibly asymptomatic) cases, caused by autochthonous transmission; (iii) a major outbreak affecting a significant proportion of the host population.

In 2007, CHIKV was identified as the pathogen responsible for the Italian outbreak only two months after the index case was recorded^3,12^, while first symptomatic CHIKV cases in 2017 occurred three months before the outbreak was detected^17^. Indeed, only a fraction of CHIKV cases are symptomatic and only a fraction of these are notified to public authorities. In particular, it is not unlikely that the first case goes unnoticed and only the accumulation of cases leads to the identification of the responsible pathogen. In our simulations, infected cases become symptomatic with probability equal to 0.82 and are notified with probability 0.54^29^ and potential delays in the detection of cases are taken into account by considering the distribution of the time lag between the arrival of imported cases and their notification to public health authorities^34^. Finally, in our model, vector control activities are assumed to be carried out as soon as the first symptomatic CHIKV case is notified, as required by the National Plan of arboviruses surveillance and control^35^.

Finally, the robustness of model outcomes was assessed for alternative climatic conditions by increasing and decreasing temperatures recorded in 2007 by 0.5, 1 and 1.5 °C. Details can be found in the Appendix S1.

### Economic model

The effectiveness of the specific intervention strategies was considered with respect to the scenario where no intervention was implemented. In order to quantify the disease burden due to CHIKV transmission and the sustained costs, a DALY approach was adopted within a cost-utility framework. DALYs can be thought of as a measurement of the gap between individuals’ current health status and an ideal health situation where the entire population lives to an advanced age, free of disease and disability. DALY is a function of disability weights, duration of the illness and years lost in case of premature death^36^.

**Figure 4:**
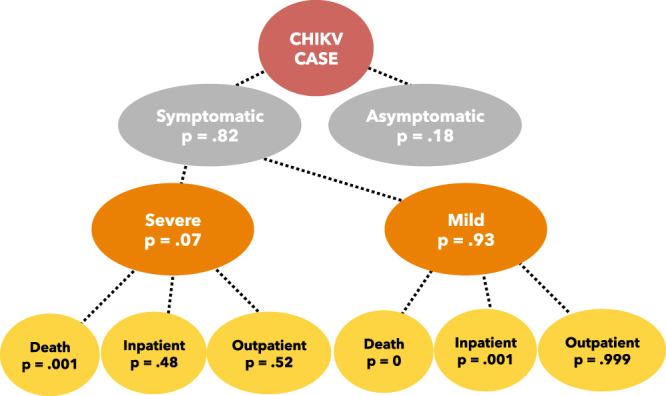
Decision tree used to classify simulated CHIKV cases. Transition probabilities to different states of the tree are reported in the circles.

Both DALYs^37^ and costs were combined with the outputs of the epidemiological model classifying each infection by means of a decision tree structured according to the probabilities of being symptomatic, severe or mild, and of showing different disease outcome including death, hospitalisation or ambulatory assistance (Fig. 4).

Costs of illness were quantified by combining treatment, hospitalisation and ambulatory costs, and the proportion of symptomatic and severe cases among simulated infected individuals. The overall costs of the considered intervention strategies (i.e. larvicides, adulticides applications and breeding sites removal) were computed taking into account also personnel-related expenditures.

DALY losses, cost of illness per case and cost of the different vector control strategies were computed using base case parameters values reported in Tables 1 and 2. Details can be found in the Appendix S1.

**Table 1:** Base case values and sampling distributions of cost of illness and DALYs loss parameters.

Model input parameter	Value (range)	Distribution for PSA	Source
***Epidemiological parameters – CHIKV***			
Proportion of symptomatic cases	0.82 (0.7, 0.9)	Truncated Normal (0.82, 0.02)	^29^
Proportion of severe cases	0.07 (0.001, 0.296)	Beta (3.69, 48.76)	Incidence data^a^
Proportion of hospitalisation among severe cases	0.48 (0.26, 0.68)	Beta (47.32, 51.5)	Incidence data^a^
Proportion of hospitalisation among mild cases	0.001 (0.000, 0.01)	Beta (0.99, 997.2)	Incidence data^a^
Proportion of deaths among severe cases	0.001 (0.000, 0.01)	Beta (0.99, 999.7)	^37^
***Cost of illness – CHIKV***			
Length of hospital stay for severe cases (in days)	4.17 (2, 7.9)	Gamma (30.86, 0.135)	Incidence data^a^
Length of hospital stay for mild cases (in days)	3.8 (1.5, 7.1)	Gamma (34.18, 0.11)	Incidence data^a^
Number of ambulatory visits per symptomatic patients	2	Point estimate	Expert opinion^b^
Cost per ambulatory visit (in euro)	47.5 (40, 55)	Triangular	Expert opinion^b^
Treatment and test cost for each ambulatory case (in euro)	328.4 (250, 406.8)	Triangular	Expert opinion^b^
Treatment and test cost for each hospitalised case (in euro)	1534 0.5 (1400, 1670)	Triangular	Expert opinion^b^
Hospital stay cost per day (in euro)	391.7 (370, 413.4)	Triangular	Expert opinion^b^
***Burden of disease – CHIKV***			
Duration of illness for each severe cases (in days)	3.2 (0.07, 15.13)	Gamma (3.14, 1.02)	Incidence data^a^
Duration of illness for each mild cases (in days)	2.9 (0.05, 13.75)	Gamma (2.58, 1.13)	Incidence data^a^
Disability weights for each severe case	0.428 (0.38, 0.47)	Beta (1047.4, 1399.7)	^37^
Disability weights for each mild case	0.195 (0.16, 0.24)	Beta (305.9, 1262.84)	^37^
Years life lost in case of death (*YLL)*	20 (0, 40)	Uniform	^37^

^a^Incidence data were obtained by^34^.

^b^Department of Infectious Diseases of the San Matteo hospital in Pavia, Italy.

**Table 2:** Base case values and sampling distributions of the costs of interventions.

	Intervention parameters		
Number of working hours per breeding sites removal on private territory (per ha)	3.04 (1.94, 4.63)	Gamma	Estimates based on field experience*
Personnel cost per breeding sites removal on private territory (per hour)	15.25 (14.76, 16)	Triangular	Estimates based on field experience^*^
Cost of breeding sites removal on public territory (per ha)	9.02 (8, 10)	Triangular	Estimates based on field experience^*^
Cost of one larvicide application on public territory (per ha)	3.73 (1.2, 8.4)	Triangular	Estimates based on field experience^*^ and^28^
Adulticide cost (per hour)	95 (90, 100)	Uniform	Estimates based on field experience^**^
Treated hectares (per hour)	8 (2, 14)	Uniform	Estimates based on field experience^**^

^*^Vector control activities performed during 2015 in the province of Trento and Belluno by the personnel of the Istituto Zooprofilattico Sperimentale delle Venezie and the Fondazione Edmund Mach (see Appendix S1 for detailed information).

^**^Vector control activities performed during 2015 in the province of Belluno by the personnel of Entostudio.

The perspective adopted for the economic analysis was that of the Italian public healthcare system. The most cost-effective intervention was assessed through the maximisation of the NHB^38^, defined as the difference between the DALYs averted and the incremental cost due to the intervention, the latter divided by the WTP value. In order to find highly cost-effective interventions, as suggested by WHO^39^, we set this value to be approximately equal to the Gross Domestic Product (GDP) per capita of Italy in 2007, namely €30000^40^. A sensitivity analysis on the WTP can be found in the Appendix S1.

### Probabilistic sensitivity analysis

Possible uncertainties in defining the optimal intervention strategy emerge as a consequence of (i) the intrinsic stochasticity of events such as the date at arrival of imported cases, the transmission process and the delay in the notification, (ii) the variability characterising model estimates as driven by the uncertainty on the epidemiological parameters, (iii) the uncertainty around estimates on costs and DALY losses. In order to account for all these factors and to reproduce different epidemiological conditions, we performed a probabilistic sensitivity analysis based on the repeated run of model simulations for each considered intervention. The proportion of simulations in which a given strategy reached the highest NHB was used to compute the probability of being the most cost-effective. Details on the procedure to assess the optimal strategy in the different epidemiological scenarios are provided in the Appendix S1.

### Availability of data and material

All data are available as referenced in the article.

## Electronic supplementary material

Appendix S1

## References

[1] Fischer D, Thomas SM, Neteler M, Tjaden NB, Beierkuhnlein C (2016). Climatic suitability of *Aedes albopictus* in Europe referring to climate change projections: comparison of mechanistic and correlative niche modelling approaches. Euro Surveillance..

[2] Guzzetta, G. *et al*. Assessing the potential risk of Zika virus epidemics in temperate areas with established *Aedes albopictus* populations. *Euro Surveillance*. **21**(15) (2016). 10.2807/1560-7917.ES.2016.21.15.30199 27104366

[3] Poletti P (2011). Transmission potential of CHIKV virus and control measures: the case of Italy. PLoS One..

[4] Guzzetta G (2016). Potential risk of dengue and chikungunya outbreaks in northern Italy based on a population model of *Aedes albopictus* (diptera: Culicidae). PLoS Neglected Tropical Diseases..

[5] Soumahoro MK (2011). The CHIKV epidemic on La Reunion Island in 2005–2006: a cost-of-illness study. PLoS Neglected Tropical Diseases..

[6] Reiter P, Fontenille D, Paupy C (2006). *Aedes albopictus* as an epidemic vector of CHIKV virus: another emerging problem. The Lancet infectious diseases..

[7] CDC, Chikungunya Virus., https://www.cdc.gov/chikungunya/ (2017).

[8] Schilte C (2013). CHIKV Virus-associated Long-term Arthralgia: A 36-month Prospective Longitudinal Study. PLoS Neglected Tropical Diseases..

[9] Weaver SC, Lecuit M (2015). CHIKV virus and the global spread of a mosquito-borne disease. New England Journal of Medicine..

[10] Seyler T (2010). Estimating the burden of disease and the economic cost attributable to CHIKV, Andhra Pradesh, India, 2005–2006. Transactions of the Royal Society of Tropical Medicine and Hygiene..

[11] Cardona-Ospina JA, Villamil-Gómez WE, Jimenez-Canizales CE, Castañeda-Hernández DM, Rodríguez-Morales AJ (2015). Estimating the burden of disease and the economic cost attributable to CHIKV, Colombia, 2014. Transactions of The Royal Society of Tropical Medicine and Hygiene..

[12] Rezza G (2007). Infection with CHIKV virus in Italy: an outbreak in a temperate region. The Lancet..

[13] ECDC, Rapid Risk Assessment: Cluster of autochthonous chikungunya cases in France (2017).

[14] Burt FJ, Rolph MS, Rulli NE, Mahalingam S, Heise MT (2012). Chikungunya: a re-emerging virus. The Lancet..

[15] ECDC, Clusters of autochthonous chikungunya cases in Italy. Rapid Risk Assessment, https://ecdc.europa.eu/en/publications-data/rapid-risk-assessment-clusters-autochthonous-chikungunyacases-italy (2017).

[16] Istituto Superiore di Sanità, Bulletin of Chikungunya outbreak., www.salute.gov.it/portale/temi/documenti/chikungunya/bollettino_chikungunya_ULTIMO.pdf (2018).

[17] Manica, M., *et al*. Transmission dynamics of the ongoing chikungunya outbreak in Central Italy: from coastal areas to the metropolitan city of Rome, summer 2017. *Eurosurveillance*, **22**(44) (2017). 10.2807/1560-7917.ES.2017.22.44.17-00685 PMC5710132 29113629

[18] Burt FJ (2017). Chikungunya virus: an update on the biology and pathogenesis of this emerging pathogen. The Lancet Infectious Diseases..

[19] Erasmus JH (2017). A chikungunya fever vaccine utilizing an insect-specific virus platform. Nature medicine..

[20] Erguler K (2017). A large-scale stochastic spatiotemporal model for *Aedes albopictus*-borne chikungunya epidemiology. PLoS One..

[21] Baldacchino F (2017). An integrated pest control strategy against the Asian tiger mosquito in northern Italy: a case study. Pest Management Science..

[22] Canali M, Rivas-Morales S, Beutels P, Venturelli C (2017). The Cost of Arbovirus Disease Prevention in Europe: Area-Wide Integrated Control of Tiger Mosquito, *Aedes albopictus*, in Emilia-Romagna, Northern Italy. International journal of environmental research and public health.

[23] Krishnamoorthy K, Harichandrakumar KT, Kumari AK, Das LK (2009). Burden of CHIKV in India: estimates of disability adjusted life years (DALY) lost in 2006 epidemic. Journal of vector borne diseases..

[24] Thuilliez J, Bellia C, Dehecq JS, Reilhes O (2014). Household-level expenditure on protective measures against mosquitoes on the island of la Réunion, France. PLoS Neglected Tropical Diseases..

[25] Guzzetta G (2017). Effectiveness and economic assessment of routine larviciding for prevention of chikungunya and dengue in temperate urban settings inEurope. PLoS Neglected Tropical Diseases..

[26] Luz PM, Vanni T, Medlock J, Paltiel AD, Galvani AP (2011). Dengue vector control strategies in an urban setting: an economic modelling assessment. The Lancet.

[27] Alfaro-Murillo JA (2016). A cost-effectiveness tool for informing policies on Zika virus control. PLoS neglected tropical diseases,.

[28] Canali, M., Controllo della zanzara tigre: analisi dei costi sostenuti dagli Enti locali., http://salute.regione.emilia-romagna.it/documentazione/rapporti/contributi/contributi_73_zanzara_tigre.pdf/view (2012).

[29] Moro ML (2010). CHIKV virus in North-Eastern Italy: a seroprevalence survey. The American journal of tropical medicine and hygiene..

[30] ECDC, Aedes albopictus - current known distribution: September 2017., https://ecdc.europa.eu/sites/portal/files/images/mosquitoes-maps-Aedes-albopictus-April-2017.jpg (2017).

[31] ECDC, Development of Aedes albopictus risk maps., http://www.epicentro.iss.it/problemi/zanzara/epid.asp (2009).

[32] ECDC, Vector control with a focus on *Aedes aegypti* and *Aedes albopictus* mosquitoes: literature review and analysis of information, https://ecdc.europa.eu/en/publications-data/vector-control-focus-aedes-aegypti-and-aedes-albopictusmosquitoes-literature (2017).

[33] European Chemical Agency, Transitional Guidance on Efficacy Assessment for Product Type 18, Insecticide, Acaricides & other Biocidal Products against Arthropods and Product Type 19, Repellents & Attractants (2017).

[34] Ministero della Salute, Sorveglianza dei casi umani di Chikungunya, Dengue, West Nile Disease ed altre arbovirosi e valutazione del rischio di trasmissione in Italia., http://www.epicentro.iss.it/problemi/westNile/pdf/Circolare_arbovirosi_2015.pdf (2015).

[35] Ministero della Salute, Piano nazionale di sorveglianza e risposta alle arbovirosi trasmesse da zanzare (aedes sp.) con particolare riferimento ai virus chikungunya, dengue e zika, http://www.salute.gov.it/portale/news/p3_2_1_1_1.jsp?lingua=italiano&menu=notizie&p=dalministero&id=3013 (2017).

[36] Anand S, Kara H (1997). Disability-adjusted life years: a critical review. Journal of health economics..

[37] LaBeaud AD, Bashir F, King CH (2011). Measuring the burden of arboviral diseases: the spectrum of morbidity and mortality from four prevalent infections. Population health metrics..

[38] Stinnet, A. & Mullay, J. Net Health Benefits: A New Framework for the Analysis of Uncertainty in Cost-Effectiveness Analysis. *Medical Decision Making*, **18**(2suppl), pp. S68–S80 (1998). 10.1177/0272989X98018002S09 9566468

[39] World Health Organization. The world health report 2002: reducing risks, promoting healthy life, http://www.who.int/whr/2002/en/ (2002). 10.1080/1357628031000116808 14741909

[40] Istituto Nazionale di Statistica, Serie storica PIL., http://dati.istat.it/Index.aspx (2017).

