# Supplementary material for: The containment of potential outbreaks triggered by imported Chikungunya cases in Italy: a cost utility epidemiological assessment of vector control measures
Source: Sci Rep. 2018 Jun 13;8:9034. doi: 10.1038/s41598-018-27443-9 (PMC5998040; doi:10.1038/s41598-018-27443-9)
Supplement: Supplementary file 1 — Appendix S1 [file 41598_2018_27443_MOESM1_ESM.pdf]

Supplementary Material

The containment of potential outbreaks triggered by imported Chikungunya cases in Italy: a cost utility epidemiological assessment of vector control measures

F. Trentini[1], P. Poletti[1, 2], F. Baldacchino[3], A. Drago[4], F. Montarsi[5], G. Capelli[5], A. Rizzoli[4], R. Rosà[4], C. Rizzo[6] , S. Merler[2] and A. Melegaro[1, 7]

[1] DONDENA Centre for Research on Social Dynamics, Bocconi University, Milan, Italy
[2] Center for Information Technology, Bruno Kessler Foundation, Trento, Italy
[3] Department of Biodiversity and Molecular Ecology, Research and Innovation Centre, Fondazione Edmund Mach, San Michele all'Adige (Trento), Italy
[4] Entostudio, Ponte San Nicolò (Padova), Italy
[5] Istituto Zooprofilattico Sperimentale delle Venezie, Padova, Italy
[6] Department of Infectious Disease, Istituto Superiore di Sanità, Rome, Italy
[7] Department of Policy Analysis and Public Management, Bocconi University, Milan, Italy

1. Model structure

The adopted epidemiological model accounts for both the transmission of pathogen between humans and mosquitoes, taking explicitly into account for the mosquito vital dynamics. The model was originally developed in [1] and extended here to simulate the impact of different control measures in response to the notification of a Chikungunya case returning from an endemic region. The transmission of Chikungunya virus is simulated in a urban area of approximately 70 ha and 4000 individuals. The human population is assumed to be constant, while temporal variation of the abundance of female adult mosquitoes is explicitly modeled.

The population dynamics of *Ae. albopictus*, which is the mosquito species representing the competent vector for the disease transmission, is modeled by considering transitions between four different life stages of the mosquito: eggs (E), larvae (L), pupae (P) and female adults (A). Variations in the mosquito abundance are simulated according to the stochastic model associated with the following system of differential equations:

$$\begin{cases} \dot{E} = n_E d_A A(1 - \frac{E}{K_E}) - (m_E + d_E)\, E \\ \dot{L} = d_E E - (m_L + d_L)L \\ \dot{P} = d_L L - (m_P + d_P)P \\ \dot{A} = \frac{1}{2} d_P P - m_A A \end{cases} \qquad \text{(Eq S1)}$$

where d_E, d_P, d_L, d_A represent the developmental rates driving eggs hatching (d_E), pupation (d_P), adult emergence (d_L) and the female adult gonotrophic cycle (d_A); m_E, m_P, m_L, m_A are the mortality rates for eggs, pupae, larvae and adults respectively. Both developmental and mortality rates are considered as temperature dependent. Finally, n_E and K_E are the average number of eggs laid in one oviposition and the carrying capacity for eggs, while the term 1/2 accounts for the 1:1 sex ratio [2].

The transmission mechanism driving the spread of the infection in the considered population is based on a SEI-SEIR schema [1]. Specifically, female adults of *Ae. albopictus* are divided in susceptible (A_S) latent (A_E) and infectious (A_I) mosquitoes; human hosts are divided in individuals who are susceptible (S_H), latent (E_H), infectious (I_H), and recovered (R_H). Briefly, susceptible vectors get infected after biting an infectious host and become infectious after a period of $1/\omega_V$ (extrinsic incubation period). Infected mosquitoes are assumed to remain infectious throughout the rest of their life. Susceptible humans get infected thought bites of infectious mosquitoes and become infectious after a period of $1/\omega_H$. Infected hosts recovers after an average infectious period of $1/\gamma$, gaining life-long immunity against reinfection. Transitions across different epidemiological classes simulated according to the stochastic model associated with the following system of differential equations:

$$\begin{cases} \dot{A_s} = \frac{1}{2} d_P P - (m_A + \lambda_V)\, A_s \\ \dot{A_E} = -m_A A_E + \lambda_V A_s - \omega_V A_E \\ \dot{A_I} = -m_A A_I + \omega_V A_E \\ \dot{S_H} = -\lambda_H S_H \\ \dot{E_H} = \lambda_H S_H - \omega_H E_H \\ \dot{I_H} = p_S \omega_H E_H - \gamma I_H \\ \dot{R_H} = \gamma I_H \end{cases} \qquad \text{(Eq. S2)}$$

where λ_V and λ_H are the force of infection on mosquitoes and on humans respectively, defined as:

$$\lambda_V = k\, \chi_V \frac{I_H}{N} \quad \text{(Eq S3)}$$

$$\lambda_H = k\, \chi_H \frac{A_I}{N} \quad \text{(Eq S4)}$$

where k represent the mosquito biting rate on humans, χ_V and χ_H are the vector and human susceptibility to the virus respectively.

In the proposed analysis parameters values driving the population dynamics described in system Eq. S1 and the epidemiological transitions described in system Eq. S2 were taken by estimates obtained in [1], where the model was calibrated on CHIKV cases notified during the outbreak occurred in Emilia-Romagna in 2007. In particular, as in [1], the epidemiological conditions simulated in our analysis reflect a basic reproduction number of 3.3 (95%CI 1.8-6) between June 21 and July 26 2007.
In our baseline scenario, consistently with previous studies [1], we assumed that breeding sites removal reduce by 40% eggs, larvae and pupae, and decrease the carrying capacity associated with the aquatic stages by 40% for 30 days. The reduction of larvae and adults through larvicides and adulticide applications are respectively assumed to be 90% and 95%. While adulticides are effective for one day, the reduction of larvae determined by larvicides is assumed to persist for one month after the intervention is carried out. Further details on model structure, calibration and parameterization can be found in [1].

2. Cost of illness and DALY losses

Cost of illness (COST) was computed according to the following equation:

$$\begin{aligned} COST \;=\; p_{sym}[p_{sev}(&{p_{amb}}^{sev}(n_{visit} cost_{visit} + {cost_{treat}}^{amb}) \\ &+ {p_{hosp}}^{sev}\big({n_{days}}^{sev} {cost_{stay}}^{hosp} + {cost_{treat}}^{hosp}\big)) \\ &+ \;(1 - p_{sev)})({p_{amb}}^{mild}(n_{visit} cost_{visit} + {cost_{treat}}^{amb}) \\ &+ \;{p_{hosp}}^{mild}\big({n_{days}}^{mild} {cost_{stay}}^{hosp} + {cost_{treat}}^{hosp}\big))] \end{aligned}$$

where p_{sym}, p_{sev} are the probabilities that a Chikungunya infected individual is symptomatic and shows severe symptoms when symptomatic; ${p_{amb}}^{sev}$, ${p_{amb}}^{mild}$, ${p_{hosp}}^{sev}$, ${p_{hosp}}^{mild}$ are respectively the probabilities that a Chikungunya symptomatic case is an ambulatory patient, an hospitalized patient for severe and mild

cases; n_{visit} is the average number of visits required for an ambulatory case; $n_{days}{}^{sev}$ and $n_{days}{}^{mild}$ are the number of days of hospital stay for hospitalized severe and mild cases respectively; $cost_{visit}$, $cost_{treat}{}^{amb}$ are respectively the costs for one visit and of all treatments and tests costs for an ambulatory patient; $cost_{treat}{}^{hosp}$ and $cost_{stay}{}^{hosp}$ are the costs of hospital stay per day and the treatments and tests costs for any hospitalized case.

The burden of disease in terms of Disability Adjusted Life Years (DALY) was computed according to the following equation:

$$DALY = p_{sev}(p_{death}{}^{sev}\,YYL + (1 - p_{death}{}^{sev})dw_{sev}n_{day\_ill}{}^{sev}) + (1 - p_{sev})dw_{mild}n_{day\_ill}{}^{mild}$$

where $p_{death}{}^{sev}$ is the probability of death for severe cases, YYL is the years life lost due to Chikungunya infection, dw_{sev} and dw_{mild} are the disability weights as reported by the Global Burden of Disease report [5] for severe and mild cases of Chikungunya infection; $n_{day\_ill}{}^{sev}$ and $n_{day\_ill}{}^{mild}$ represent the average duration of illness for severe and mild cases.
Cost parameters were derived from analyses of national records routinely collected at hospital discharge of patients (Hospital Discharge Records), HDR at the San Matteo hospital in Pavia, Italy, complemented with the expert opinion provided by doctors from the Department of Infectious Diseases of the same hospital.
Collected data consisted of routine health data and medical records, which were encrypted and anonymous, and did not contain any information that might be used to identify individual patients; therefore, the study did not require informed consent.
Computed costs for ambulatory visits are based on an average of 2 visits per patient, with a general practitioner (GP) cost of 47.5 euros (range 45-50) per visit and tests/treatment costs of 328.4 euros (range 250-407). The cost of per day of hospitalization for one case resulted 391.7 euros (range 370-413) and the average cost for tests/treatments performed during the hospital stay resulted 1534.5 euros per case (range 1400-1670).
Parameters to compute DALYs per Chikungunya case were either derived from incidence records of imported Chikungunya cases (p_{sev}, p_{hosp}, $n_{day}{}^{sev}$, $n_{day}{}^{mild}$, $n_{day\_ill}{}^{sev}$ and $n_{day\_ill}{}^{mild}$) or obtained from literature [6,7].
The overall cost per Chikungunya case was estimated to be €424.9 (95% CI: 280.4, 795.5) and its relative burden in terms of DALYs 0.45 years (95% CI: 0.01, 2.57). Estimated distributions of such quantities are shown in Fig. S1

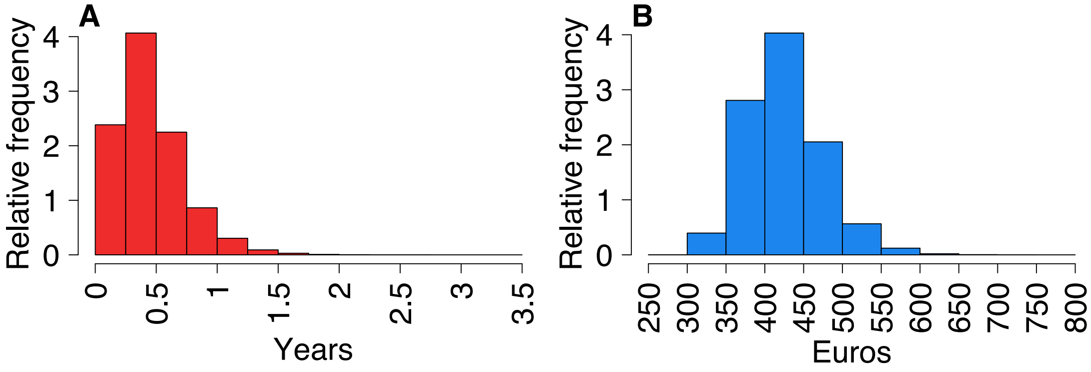

Fig. S1 **A** Distribution of DALY losses per CHIKV case. **B** Distribution of sustained cost per CHIKV case

3. Intervention strategies and vector control activities

The CHIKV outbreak occurred in Italy during the 2007 was triggered by the return of an infectious traveler on June 23rd. Once the CHIKV virus was found to be the pathogen responsible of the epidemic on August 23rd (i.e., two months after the arrival of the first case), sudden measures were taken to reduce the abundance of *Ae. albopictus*. These included breeding sites removal and larvicide applications in drains and adulticide applications. The latter was conducted for 3 consecutive days (from August 23rd to 25th). In our baseline scenario, we assumed breeding sites removal (B) to reduce by 40% eggs, larvae and pupae, and decrease the carrying capacity associated with the aquatic stages by 40% for 30 days. The reduction of larvae and adults through larvicide (L) and adulticide (A) applications are respectively assumed to be 90% and 95%. Each adulticide application is assumed to be effective for one day, while the reduction of larvae determined by larvicide treatments is assumed to persist for one month after the intervention is carried out.
The cost of the implemented program is obtained by summing up the costs of single vector control activities, which are computed as follows:

$$COST_B = n_{ha}(n_{hours}cost_{bs\_pers} + cost_{bs\_pub})$$

$$COST_L = n_{ha}cost_{larv\_ha}$$

$$COST_A = n_{days}\frac{n_{ha}}{n_{treat\_ha\_hour}}cost_{adult\_hour}$$

where n_{ha} is the number of hectare treated, n_{hours} and $cost_{bs\_pers}$ are the number of working hours per hectare and the personnel cost per hour for breeding sites removal on private territory; $cost_{bs}$ represents the sanitation cost per hectare on public territory. As for larvicides, $cost_{larv\_ha}$ is the cost of larvicide applications on public territory per hectare. Finally, $cost_{adult\_hour}$ is the cost per adulticide application per hour, including both personnel and product costs; $n_{treat\_ha\_hour}$ is the number of hectares treated with adulticides per hour and n_{days} is the number of days for which adulticide applications are conducted.

4. Estimates of intervention costs based on field experience

Interventions costs were estimated from vector control activities carried out in several municipalities in the provinces of Belluno and Trento during the summer 2015 [8]. Larvicides were applied monthly in public catch basins using granules of *Bacillus thuringiensis israelensis* (*Bti*) and *B. sphaericus* (Vectomax®; Valent BioSciences, Libertyville, IL) at a dose of 10 g per catch basin according to supplier recommendations. Furthermore, *Bti* larvicide tablets (Vectobac® DT; Valent BioSciences, Libertyville, IL) were freely distributed to citizens through a door-to-door campaign to treat private catch basins. During the door-to-door campaign, citizens were informed on mosquito control measures using educational materials (e.g. brochure), and potential breeding sites were removed. All these interventions were conducted by skilled technicians with expertise in entomology.

Adulticides costs were provided by Entostudio after vector control activities had been performed during 2015 in the province of Belluno by its personnel.

The resulting cost per hectare for breeding sites removal is €19.63 (95% CI: 5.77, 45.66), for larvicides €3.73 (95% CI: 1.2, 8.4) and for adulticides €15.43 (95% CI: 6.43, 49.94). Distributions of intervention costs per hectare are shown in Fig. S2.

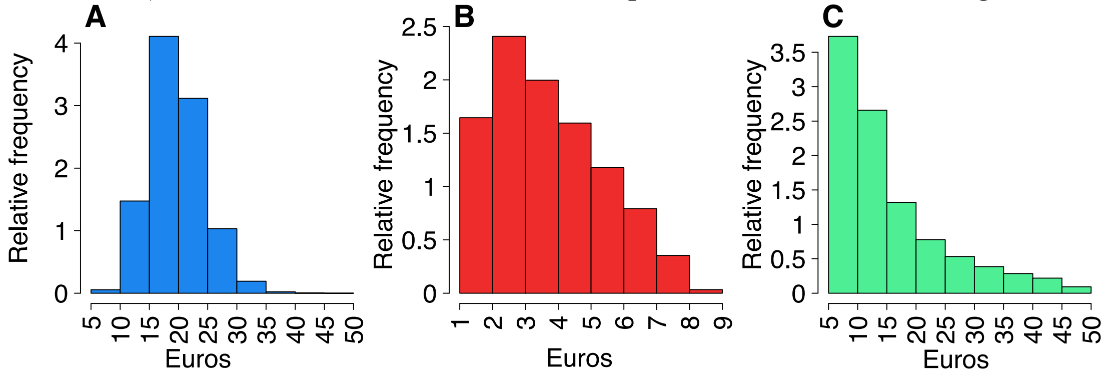

Fig. S3 Distribution of cost sustained per hectare for breeding site removal (A), larvicide applications (B) and adulticide applications (C).

5. Cost-effectiveness analysis framework

The net health benefit (NHB) [9] was derived for each intervention. The uncertainty surrounding our estimates of the NHB was taken into consideration through random samples from the distributions presented above, and by considering the variability characterizing incidence estimates provided by model simulations. Specifically, for each model realization the NHB is computed as

$$NHB = \Delta D - \Delta C/k$$

where ΔD and ΔC are respectively DALY averted and incremental costs due to intervention, k represents the willingness to pay, which is defined by the amount of money the healthcare system is willing to pay for one DALY averted in the population. The value of k was fixed at 30K euros, roughly representing the GDP per capita of Italy in 2007.

We simulate different epidemiological scenarios in which the first notification occurs in different periods during the mosquito season. These were obtained by sampling the dates of arrival and possible notification delays from the corresponding distributions observed for imported CHIKV cases in Italy in the last ten years. Simulations were grouped in two-weeks periods from June to October, according to simulated dates of notification of the first case. For each scenario, 10,000 stochastic realizations of the NHB were obtained by combining 100 stochastic model realizations with 100 possible configuration of DALY loss per case, cost per case and cost of intervention as drawn from the estimated distributions shown in Fig S1 and Fig. S2.

For each run, an optimal strategy was defined as the one associated with the highest NHB. Probabilities of being the most cost-effective strategy was computed for each intervention considered as the fraction of simulations for which the intervention was associated with the highest NHB.

6. Sensitivity analysis

A sensitivity analysis was conducted to assess how our results on the cost-effectiveness of the considered interventions may change as we make different assumptions on parameters driving the efficacy of vector control activities, the willingness to pay on temperature records used to simulate the temporal variation in the vector abundance at different time points of the mosquito breeding season.

6.1. Temperatures

We considered illustrative temperature patterns by decreasing and increasing all temperatures observed in 2007 in Emilia Romagna [1] by 0.5°C, 1°C, and 1.5°C, therefore assuming a constant variation over time.
Obtained results show that the cost-effectiveness of interventions is sensitive to changes in temperatures between seasons.

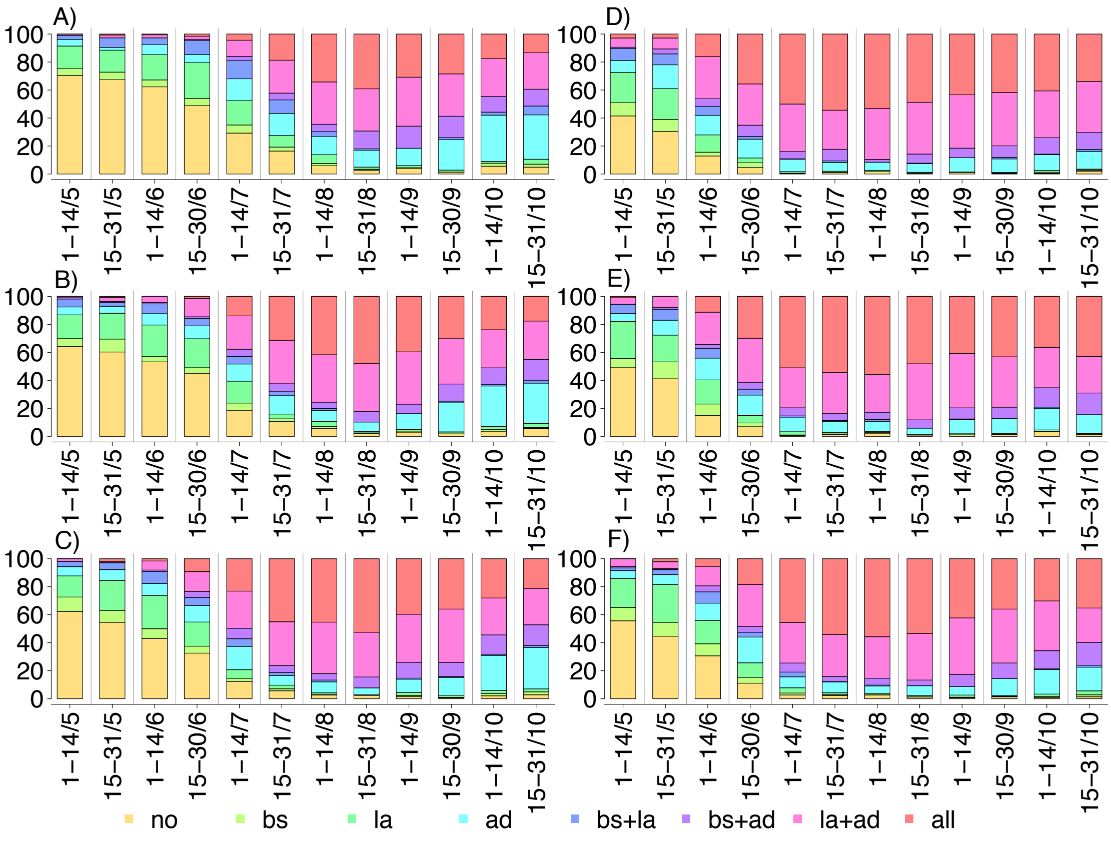

Fig. S4 The six panels show probabilities of cost effectiveness of all possible strategies for different times at first notifications with temperatures decreased by 1.5, 1 or 0.5°C (respectively panels A, B and C) or increased by 0.5, 1 or 1.5°C (respectively panels D, E and F).

Fig. S4 shows that the probability of cost effectiveness of the non-intervention decreases as temperatures increase, while the cost-effectiveness of the integrated (ie. combined) intervention increases. Our results suggest that in colder seasons (panel A and B) it would be beneficial to not intervene if a Chikungunya case is reported before mid-June, while later in the season adulticides emerge as the optimal strategy. During warmer seasons (panel E and F) reactive interventions are cost-effective since the beginning of the mosquito breeding season. In this particular case, the best intervention strategy would be represented either by the

combination of adulticides and larvicides alone, or with the addition of breeding sites removal.

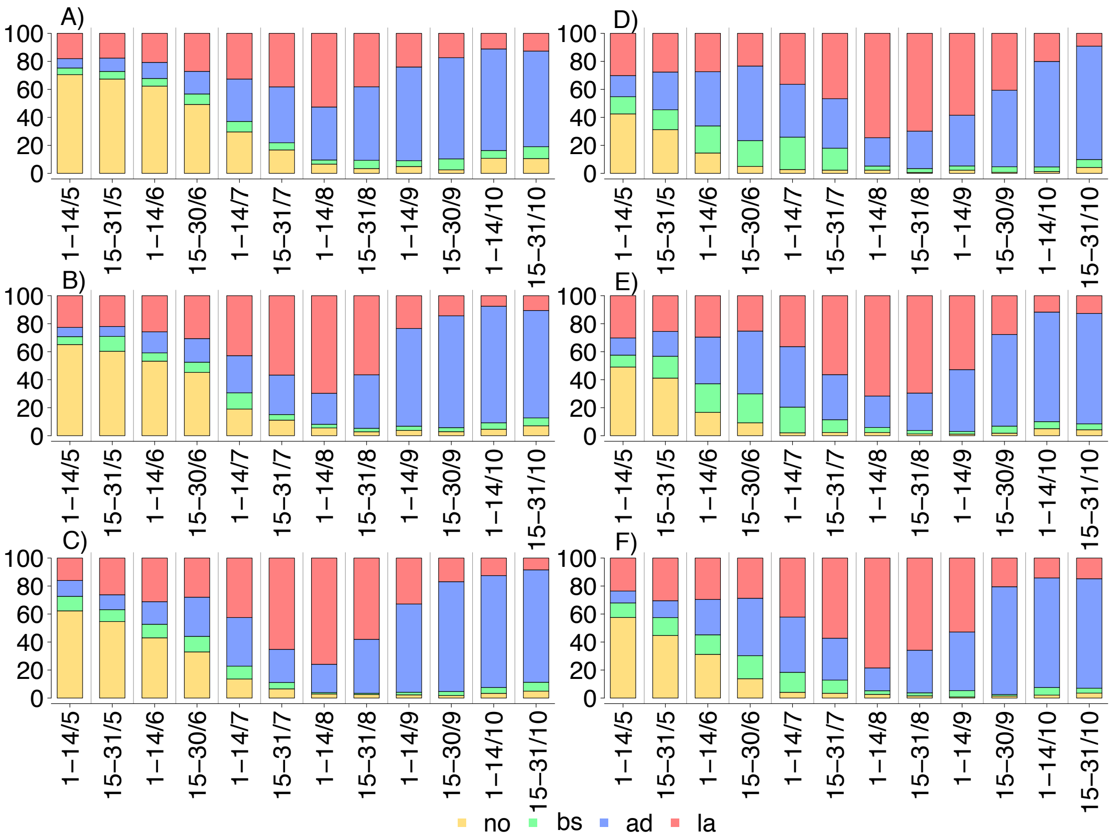

Fig. S5 The six panels show probabilities of cost effectiveness of single strategies for different times at first notifications with temperatures decreased by 1.5, 1 or 0.5°C (respectively panels A, B and C) or increased by 0.5, 1 or 1.5°C (respectively panels D, E and F).

Fig. S5 shows results obtained when single vector control activities are compared. Obtained results suggest that, colder temperatures increase the cost effectiveness of adulticides in respose to notification of CHIKV in the middle or towards the end of the mosquito season, as a consequence of their immediate impact on the vector abundance through the reduction of female adults of Ae. albopictus. On the opposite, warmer temperatures increase benefits coming from larvicide applications, which are characterized by a more long lasting effect on the mosquito population density. However, in warmer seasons, adulticides would be the most cost effective strategy in response to notifications occuring in late spring and early summer.

6.2. Efficacy of interventions

We tested the robustness of obtained results on the cost-effectiveness of interventions in our baseline analysis, by considering the uncertainty surrounding estimates of the efficacy associated with vector control activities. Cost-effectiveness results were therefore computed by sampling the efficacy of different vector control activities from uniform distributions respectively in the ranges 20-60% for breeding sites removal, and 60-95% for larvicide and adulticide applications.

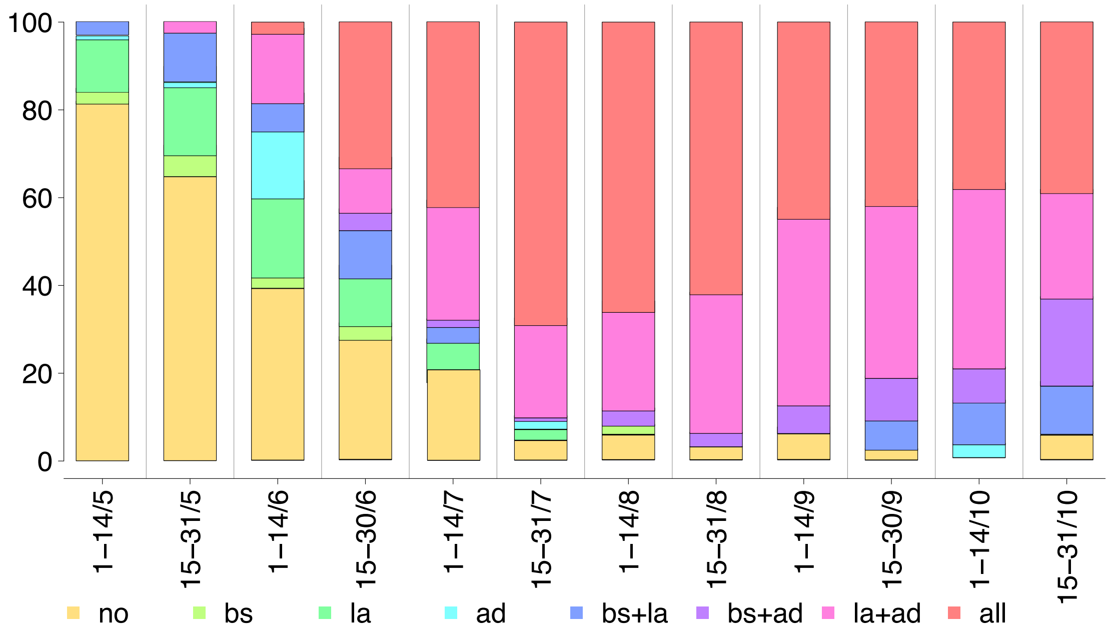

Fig. S6 Probabilities of cost effectiveness of all possible strategies for different times at first notifications, accounting for variability in the efficacy of interventions

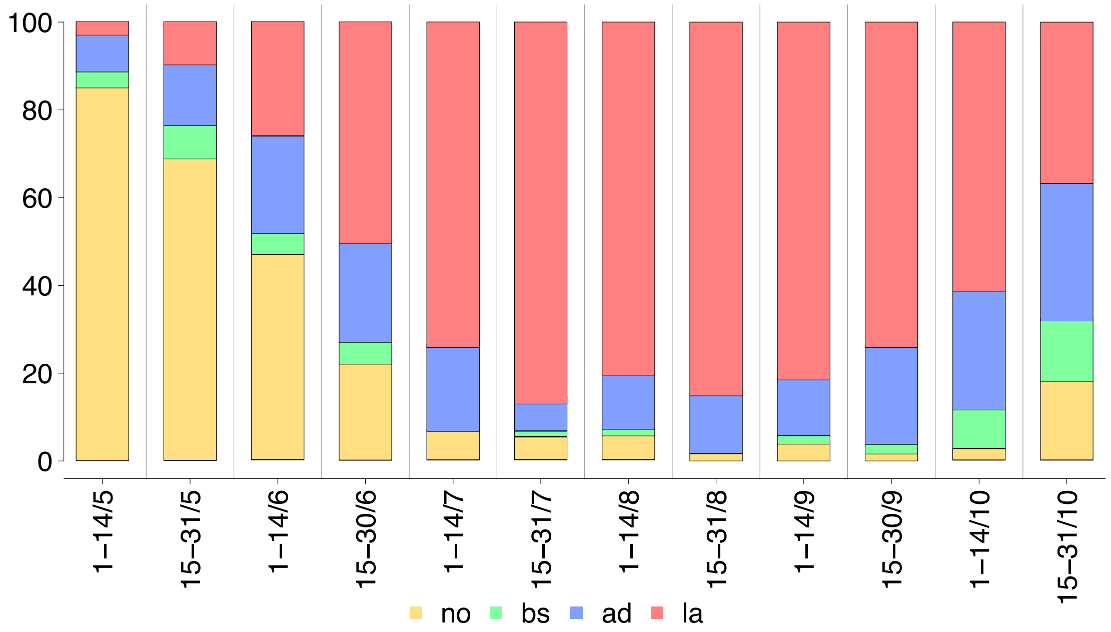

Fig. S7 Probabilities of cost effectiveness of single interventions for different times at first notifications, accounting for variability in the efficacy of interventions

Although a slightly higher probability of cost effectiveness for the non intervention, due to a broader range of efficacies accounted for in the sensitivity analysis, obtained results are robust with respect to assumption on efficacy of vector control activities made in our baseline analysis (see Fig. S6).

6.3. Willingness to pay

A sensitivity analysis was finally conducted by assuming that the willingness to pay was either €20K or €40K. The probabilities of cost-effectiveness of interventions will not be affected by assumptions on the willingness to pay, since when they prove to be cost-effective they also result to be cost-saving.

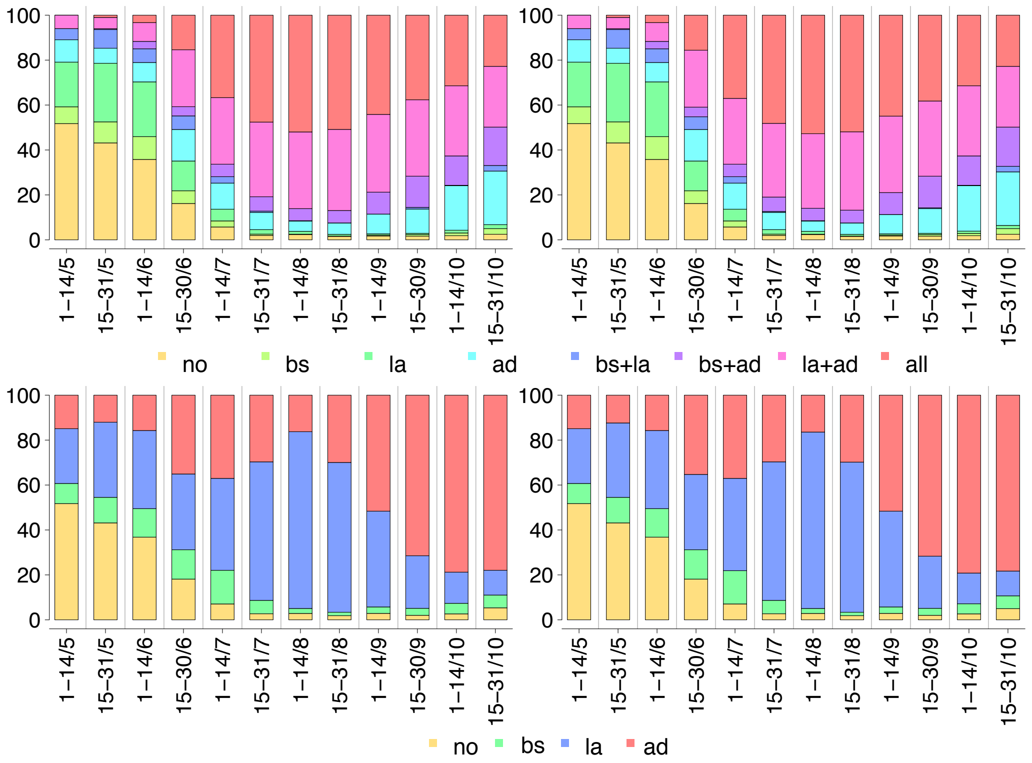

Fig. S8 Probabilities of cost effectiveness assuming different values for the willingness to pay: €20K for all strategies (A) and for single interventions (C), and €40K for all strategies (B) and for single interventions (D)

Indeed, as shown in Fig. S8, no significant differences emerge when the value for the willingness to pay is set to €20K or €40K, both when all possible combinations of the vector control activities are considered and when single interventions are considered separately.

References

[1] Poletti P, Messeri G, Ajelli M, Vallorani R, Rizzo C, Merler S. Transmission potential of CHIKV virus and control measures: the case of Italy. *PLoS One* 2011; **6**(5): e18860

[2] Delatte,H, Gimonneau G, Triboire A, Fontenille D. Influence of temperature on immature development, survival, longevity, fecundity, and gonotrophic cycles of Aedes albopictus, vector of chikungunya and dengue in the Indian Ocean. *Journal of medical entomology* 2009; **46**(1): 33-41

[3] Anderson R, May RM. Infectious diseases of humans: dynamics and control. Oxford, UK: Oxford University Press 1992

[4] Rezza G et al. Infection with CHIKV virus in Italy: an outbreak in a temperate region. *The Lancet* 2007; **370**(9602): 1840-1846.

[5] Murray CJ, Lopez AD. Global burden of disease. Boston: Harvard University Press 1996; **1**: 118-201

[6] LaBeaud AD, Bashir F, King CH. Measuring the burden of arboviral diseases: the spectrum of morbidity and mortality from four prevalent infections. *Population health metrics* 2011; **9**(1): 1

[7] Moro ML, et al. CHIKV virus in North-Eastern Italy: a seroprevalence survey. *The American journal of tropical medicine and hygiene* 2010; **82**(3): 508-11.

[8] Baldacchino F, et al. An integrated pest control strategy against the Asian tiger mosquito in northern Italy: a case study. *Pest Management Science* 2017; **73**(1): 87-93.

[9] Stinnet A, Mullay J. Net Health Benefits: A New Framework for the Analysis of Uncertainty in Cost-Effectiveness Analysis. *NBER Technical Working Paper* 1998; **227**
